# SELF-FUNDED PERSONAL CARE IS COMMON AND COSTLY FOR PEOPLE WITH DEMENTIA ACROSS THE INCOME SPECTRUM

**DOI:** 10.1093/geroni/igae098.4304

**Published:** 2024-12-31

**Authors:** Jennifer Reckrey, Karen Shen, Yang Yang

**Affiliations:** Icahn School of Medicine at Mount Sinai, Icahn School of Medicine at Mount Sinai, New York, United States; Johns Hopkins University, Baltimore, Maryland, United States; Johns Hopkins University, Baltimore, Maryland, United States

## Abstract

remain in the community; those without Medicaid coverage or long-term care insurance must self-fund this care (i.e., pay for privately or out-of-pocket). Using data from the Health and Retirement Study (2002-2018), we identified the prevalence and financial burden of self-funded paid care among community-dwelling people ≥65 who received help with ≥1 activity of daily living (n=4666). Dementia status, receipt of paid care, and financial information (income, cost of self-funded paid care) were determined by survey responses. One-quarter of PLWD (versus 14% without dementia) used self-funded care. Among PLWD, the probability of self-funding care increased with income, but was significant for all income groups: 15%, 23%, 32%, and 48% of those with incomes 100%, 100-200%, 200-400%, and ≥400 of the federal poverty level respectively self-funded paid care. The amount and cost of this self-funded care was high, particularly for PLWD: 45% of PLWD (versus 22% without dementia) received ≥40 hours/week of self-funded care and nearly half spent ≥$1000/month self-funding paid care. The median PLWD that self-funded care spent 45% of their monthly income on self-funded care; in the lowest-income group, the median PLWD spent nearly 75% of their monthly income on self-funded paid care. Given self-funded paid care is common and costly throughout the income spectrum, programs and policies aimed at easing the financial burden of self-funded paid care (e.g., expanded access to Medicaid home and community-based services) are essential, particularly for low-income individuals and families.

